# Supplementary material for: Parametric modeling of under-5 children survival among 30 African countries: Lognormal accelerated failure time gamma shared frailty model
Source: PLoS One. 2025 Jan 24;20(1):e0314955. doi: 10.1371/journal.pone.0314955 (PMC11759998; doi:10.1371/journal.pone.0314955)
Supplement: S1 Table — (DOCX) [file pone.0314955.s001.docx]

**S1 Table**: Life table showing the survival to death among under-5 children in Africa using recent DHS from 2014–2022 (n = 226862)

| Time | Total | Fail | Lost | Function | Error | [95% Conf. Int.] |
| --- | --- | --- | --- | --- | --- | --- |
| 1 | 226862 | 2416 | 0 | 0.9894 | 0.0002 | 0.9889 0.9898 |
| 2 | 224446 | 389 | 0 | 0.9876 | 0.0002 | 0.9872 0.9881 |
| 3 | 224057 | 337 | 0 | 0.9862 | 0.0002 | 0.9857 0.9866 |
| 4 | 223720 | 118 | 0 | 0.9856 | 0.0002 | 0.9851 0.9861 |
| 5 | 223602 | 137 | 0 | 0.985 | 0.0003 | 0.9845 0.9855 |
| 6 | 223465 | 89 | 0 | 0.9846 | 0.0003 | 0.9841 0.9851 |
| 7 | 223376 | 242 | 0 | 0.9836 | 0.0003 | 0.9830 0.9841 |
| 8 | 223134 | 49 | 0 | 0.9834 | 0.0003 | 0.9828 0.9839 |
| 9 | 223085 | 32 | 0 | 0.9832 | 0.0003 | 0.9827 0.9837 |
| 10 | 223053 | 46 | 0 | 0.983 | 0.0003 | 0.9825 0.9835 |
| 11 | 223007 | 20 | 0 | 0.9829 | 0.0003 | 0.9824 0.9834 |
| 12 | 222987 | 22 | 0 | 0.9828 | 0.0003 | 0.9823 0.9833 |
| 13 | 222965 | 15 | 0 | 0.9828 | 0.0003 | 0.9822 0.9833 |
| 14 | 222950 | 129 | 0 | 0.9822 | 0.0003 | 0.9816 0.9827 |
| 15 | 222821 | 52 | 0 | 0.982 | 0.0003 | 0.9814 0.9825 |
| 16 | 222769 | 9 | 0 | 0.9819 | 0.0003 | 0.9814 0.9825 |
| 17 | 222760 | 15 | 0 | 0.9819 | 0.0003 | 0.9813 0.9824 |
| 18 | 222745 | 15 | 0 | 0.9818 | 0.0003 | 0.9812 0.9823 |
| 19 | 222730 | 7 | 0 | 0.9818 | 0.0003 | 0.9812 0.9823 |
| 20 | 222723 | 29 | 0 | 0.9816 | 0.0003 | 0.9811 0.9822 |
| 21 | 222694 | 66 | 0 | 0.9813 | 0.0003 | 0.9808 0.9819 |
| 22 | 222628 | 12 | 0 | 0.9813 | 0.0003 | 0.9807 0.9818 |
| 23 | 222616 | 7 | 0 | 0.9813 | 0.0003 | 0.9807 0.9818 |
| 24 | 222609 | 9 | 0 | 0.9812 | 0.0003 | 0.9806 0.9818 |
| 25 | 222600 | 10 | 0 | 0.9812 | 0.0003 | 0.9806 0.9817 |
| 26 | 222590 | 5 | 0 | 0.9811 | 0.0003 | 0.9806 0.9817 |
| 27 | 222585 | 7 | 0 | 0.9811 | 0.0003 | 0.9805 0.9817 |
| 28 | 222578 | 10 | 0 | 0.9811 | 0.0003 | 0.9805 0.9816 |
| 29 | 222568 | 6 | 0 | 0.981 | 0.0003 | 0.9805 0.9816 |
| 30 | 222562 | 19 | 4440 | 0.981 | 0.0003 | 0.9804 0.9815 |
| 31 | 218103 | 461 | 0 | 0.9789 | 0.0003 | 0.9783 0.9795 |
| 32 | 217642 | 373 | 0 | 0.9772 | 0.0003 | 0.9766 0.9778 |
| 33 | 217269 | 349 | 0 | 0.9756 | 0.0003 | 0.9750 0.9763 |
| 34 | 216920 | 242 | 0 | 0.9746 | 0.0003 | 0.9739 0.9752 |
| 35 | 216678 | 207 | 0 | 0.9736 | 0.0003 | 0.9730 0.9743 |
| 36 | 216471 | 275 | 0 | 0.9724 | 0.0003 | 0.9717 0.9731 |
| 37 | 216196 | 253 | 0 | 0.9712 | 0.0004 | 0.9705 0.9719 |
| 38 | 215943 | 233 | 0 | 0.9702 | 0.0004 | 0.9695 0.9709 |
| 39 | 215710 | 257 | 0 | 0.969 | 0.0004 | 0.9683 0.9698 |
| 40 | 215453 | 150 | 0 | 0.9684 | 0.0004 | 0.9676 0.9691 |
| 41 | 215303 | 145 | 0 | 0.9677 | 0.0004 | 0.9670 0.9684 |
| 42 | 215158 | 295 | 0 | 0.9664 | 0.0004 | 0.9656 0.9671 |
| 43 | 214863 | 128 | 0 | 0.9658 | 0.0004 | 0.9651 0.9666 |
| 44 | 214735 | 109 | 0 | 0.9653 | 0.0004 | 0.9646 0.9661 |
| 45 | 214626 | 98 | 0 | 0.9649 | 0.0004 | 0.9641 0.9656 |
| 46 | 214528 | 59 | 0 | 0.9646 | 0.0004 | 0.9638 0.9654 |
| 47 | 214469 | 47 | 0 | 0.9644 | 0.0004 | 0.9636 0.9652 |
| 48 | 214422 | 114 | 0 | 0.9639 | 0.0004 | 0.9631 0.9647 |
| 49 | 214308 | 34 | 0 | 0.9637 | 0.0004 | 0.9630 0.9645 |
| 50 | 214274 | 42 | 0 | 0.9636 | 0.0004 | 0.9628 0.9643 |
| 51 | 214232 | 32 | 0 | 0.9634 | 0.0004 | 0.9626 0.9642 |
| 52 | 214200 | 29 | 0 | 0.9633 | 0.0004 | 0.9625 0.9640 |
| 53 | 214171 | 26 | 0 | 0.9632 | 0.0004 | 0.9624 0.9639 |
| 55 | 214145 | 1 | 0 | 0.9632 | 0.0004 | 0.9624 0.9639 |
| 60 | 214144 | 0 | 4717 | 0.9632 | 0.0004 | 0.9624 0.9639 |
| 62 | 209427 | 492 | 0 | 0.9609 | 0.0004 | 0.9601 0.9617 |
| 63 | 208935 | 138 | 0 | 0.9603 | 0.0004 | 0.9594 0.9611 |
| 64 | 208797 | 36 | 0 | 0.9601 | 0.0004 | 0.9593 0.9609 |
| 66 | 208761 | 1 | 0 | 0.9601 | 0.0004 | 0.9593 0.9609 |
| 72 | 208760 | 1 | 0 | 0.9601 | 0.0004 | 0.9593 0.9609 |
| 78 | 208759 | 1 | 0 | 0.9601 | 0.0004 | 0.9593 0.9609 |
| 90 | 208758 | 0 | 4471 | 0.9601 | 0.0004 | 0.9593 0.9609 |
| 120 | 204287 | 0 | 4698 | 0.9601 | 0.0004 | 0.9593 0.9609 |
| 131 | 199589 | 22 | 0 | 0.96 | 0.0004 | 0.9592 0.9608 |
| 150 | 199567 | 0 | 4553 | 0.96 | 0.0004 | 0.9592 0.9608 |
| 180 | 195014 | 0 | 4717 | 0.96 | 0.0004 | 0.9592 0.9608 |
| 210 | 190297 | 0 | 4510 | 0.96 | 0.0004 | 0.9592 0.9608 |
| 240 | 185787 | 0 | 4458 | 0.96 | 0.0004 | 0.9592 0.9608 |
| 270 | 181329 | 0 | 4405 | 0.96 | 0.0004 | 0.9592 0.9608 |
| 300 | 176924 | 0 | 4084 | 0.96 | 0.0004 | 0.9592 0.9608 |
| 330 | 172840 | 0 | 4382 | 0.96 | 0.0004 | 0.9592 0.9608 |
| 360 | 168458 | 0 | 4797 | 0.96 | 0.0004 | 0.9592 0.9608 |
| 390 | 163661 | 0 | 4755 | 0.96 | 0.0004 | 0.9592 0.9608 |
| 420 | 158906 | 0 | 4515 | 0.96 | 0.0004 | 0.9592 0.9608 |
| 450 | 154391 | 0 | 4242 | 0.96 | 0.0004 | 0.9592 0.9608 |
| 480 | 150149 | 0 | 4435 | 0.96 | 0.0004 | 0.9592 0.9608 |
| 510 | 145714 | 0 | 4218 | 0.96 | 0.0004 | 0.9592 0.9608 |
| 540 | 141496 | 0 | 4145 | 0.96 | 0.0004 | 0.9592 0.9608 |
| 570 | 137351 | 0 | 3910 | 0.96 | 0.0004 | 0.9592 0.9608 |
| 600 | 133441 | 0 | 3860 | 0.96 | 0.0004 | 0.9592 0.9608 |
| 630 | 129581 | 0 | 3574 | 0.96 | 0.0004 | 0.9592 0.9608 |
| 660 | 126007 | 0 | 3711 | 0.96 | 0.0004 | 0.9592 0.9608 |
| 690 | 122296 | 0 | 3379 | 0.96 | 0.0004 | 0.9592 0.9608 |
| 720 | 118917 | 0 | 3908 | 0.96 | 0.0004 | 0.9592 0.9608 |
| 750 | 115009 | 0 | 3832 | 0.96 | 0.0004 | 0.9592 0.9608 |
| 780 | 111177 | 0 | 3539 | 0.96 | 0.0004 | 0.9592 0.9608 |
| 810 | 107638 | 0 | 3388 | 0.96 | 0.0004 | 0.9592 0.9608 |
| 840 | 104250 | 0 | 3277 | 0.96 | 0.0004 | 0.9592 0.9608 |
| 870 | 100973 | 0 | 3161 | 0.96 | 0.0004 | 0.9592 0.9608 |
| 900 | 97812 | 0 | 2984 | 0.96 | 0.0004 | 0.9592 0.9608 |
| 930 | 94828 | 0 | 2904 | 0.96 | 0.0004 | 0.9592 0.9608 |
| 960 | 91924 | 0 | 2685 | 0.96 | 0.0004 | 0.9592 0.9608 |
| 990 | 89239 | 0 | 2554 | 0.96 | 0.0004 | 0.9592 0.9608 |
| 1020 | 86685 | 0 | 2509 | 0.96 | 0.0004 | 0.9592 0.9608 |
| 1050 | 84176 | 0 | 2284 | 0.96 | 0.0004 | 0.9592 0.9608 |
| 1080 | 81892 | 0 | 2240 | 0.96 | 0.0004 | 0.9592 0.9608 |
| 1110 | 79652 | 0 | 1987 | 0.96 | 0.0004 | 0.9592 0.9608 |
| 1140 | 77665 | 0 | 2069 | 0.96 | 0.0004 | 0.9592 0.9608 |
| 1170 | 75596 | 0 | 1805 | 0.96 | 0.0004 | 0.9592 0.9608 |
| 1200 | 73791 | 0 | 1680 | 0.96 | 0.0004 | 0.9592 0.9608 |
| 1230 | 72111 | 0 | 1697 | 0.96 | 0.0004 | 0.9592 0.9608 |
| 1260 | 70414 | 0 | 1603 | 0.96 | 0.0004 | 0.9592 0.9608 |
| 1290 | 68811 | 0 | 1568 | 0.96 | 0.0004 | 0.9592 0.9608 |
| 1320 | 67243 | 0 | 1458 | 0.96 | 0.0004 | 0.9592 0.9608 |
| 1350 | 65785 | 0 | 1387 | 0.96 | 0.0004 | 0.9592 0.9608 |
| 1380 | 64398 | 0 | 1316 | 0.96 | 0.0004 | 0.9592 0.9608 |
| 1410 | 63082 | 0 | 1267 | 0.96 | 0.0004 | 0.9592 0.9608 |
| 1440 | 61815 | 0 | 1644 | 0.96 | 0.0004 | 0.9592 0.9608 |
| 1470 | 60171 | 0 | 1327 | 0.96 | 0.0004 | 0.9592 0.9608 |
| 1500 | 58844 | 0 | 1407 | 0.96 | 0.0004 | 0.9592 0.9608 |
| 1530 | 57437 | 0 | 1273 | 0.96 | 0.0004 | 0.9592 0.9608 |
| 1560 | 56164 | 0 | 1221 | 0.96 | 0.0004 | 0.9592 0.9608 |
| 1590 | 54943 | 0 | 1203 | 0.96 | 0.0004 | 0.9592 0.9608 |
| 1620 | 53740 | 0 | 1178 | 0.96 | 0.0004 | 0.9592 0.9608 |
| 1650 | 52562 | 0 | 1134 | 0.96 | 0.0004 | 0.9592 0.9608 |
| 1680 | 51428 | 0 | 1078 | 0.96 | 0.0004 | 0.9592 0.9608 |
| 1710 | 50350 | 0 | 1021 | 0.96 | 0.0004 | 0.9592 0.9608 |
| 1740 | 49329 | 0 | 1011 | 0.96 | 0.0004 | 0.9592 0.9608 |
| 1770 | 48318 | 0 | 965 | 0.96 | 0.0004 | 0.9592 0.9608 |
| 1800 | 47353 | 2 | 4.70E+04 | 0.9599 | 0.0004 | 0.9591 0.9607 |
