# Supplementary material for: Parametric modeling of under-5 children survival among 30 African countries: Lognormal accelerated failure time gamma shared frailty model
Source: PLoS One. 2025 Jan 24;20(1):e0314955. doi: 10.1371/journal.pone.0314955 (PMC11759998; doi:10.1371/journal.pone.0314955)
Supplement: S2 Table — (DOCX) [file pone.0314955.s002.docx]

**S2 Table**: Cumulative hazard function among children in Africa using recent DHS 2023(n = 226862).

| Time | Total | Fail | Lost | Failure Function | Error | [95% Conf. Int.] |
| --- | --- | --- | --- | --- | --- | --- |
| 1 | 226862 | 2416 | 0 | 0.0106 | 0.0002 | 0.0102, 0.0111 |
| 2 | 224446 | 389 | 0 | 0.0124 | 0.0002 | 0.0119, 0.0128 |
| 3 | 224057 | 337 | 0 | 0.0138 | 0.0002 | 0.0134, 0.0143 |
| 4 | 223720 | 118 | 0 | 0.0144 | 0.0002 | 0.0139, 0.0149 |
| 5 | 223602 | 137 | 0 | 0.015 | 0.0003 | 0.0145, 0.0155 |
| 6 | 223465 | 89 | 0 | 0.0154 | 0.0003 | 0.0149, 0.0159 |
| 7 | 223376 | 242 | 0 | 0.0164 | 0.0003 | 0.0159, 0.0170 |
| 8 | 223134 | 49 | 0 | 0.0166 | 0.0003 | 0.0161, 0.0172 |
| 9 | 223085 | 32 | 0 | 0.0168 | 0.0003 | 0.0163, 0.0173 |
| 10 | 223053 | 46 | 0 | 0.017 | 0.0003 | 0.0165, 0.0175 |
| 11 | 223007 | 20 | 0 | 0.0171 | 0.0003 | 0.0166, 0.0176 |
| 12 | 222987 | 22 | 0 | 0.0172 | 0.0003 | 0.0167, 0.0177 |
| 13 | 222965 | 15 | 0 | 0.0172 | 0.0003 | 0.0167, 0.0178 |
| 14 | 222950 | 129 | 0 | 0.0178 | 0.0003 | 0.0173, 0.0184 |
| 15 | 222821 | 52 | 0 | 0.018 | 0.0003 | 0.0175, 0.0186 |
| 16 | 222769 | 9 | 0 | 0.0181 | 0.0003 | 0.0175, 0.0186 |
| 17 | 222760 | 15 | 0 | 0.0181 | 0.0003 | 0.0176, 0.0187 |
| 18 | 222745 | 15 | 0 | 0.0182 | 0.0003 | 0.0177, 0.0188 |
| 19 | 222730 | 7 | 0 | 0.0182 | 0.0003 | 0.0177, 0.0188 |
| 20 | 222723 | 29 | 0 | 0.0184 | 0.0003 | 0.0178, 0.0189 |
| 21 | 222694 | 66 | 0 | 0.0187 | 0.0003 | 0.0181, 0.0192 |
| 22 | 222628 | 12 | 0 | 0.0187 | 0.0003 | 0.0182, 0.0193 |
| 23 | 222616 | 7 | 0 | 0.0187 | 0.0003 | 0.0182, 0.0193 |
| 24 | 222609 | 9 | 0 | 0.0188 | 0.0003 | 0.0182, 0.0194 |
| 25 | 222600 | 10 | 0 | 0.0188 | 0.0003 | 0.0183, 0.0194 |
| 26 | 222590 | 5 | 0 | 0.0189 | 0.0003 | 0.0183, 0.0194 |
| 27 | 222585 | 7 | 0 | 0.0189 | 0.0003 | 0.0183, 0.0195 |
| 28 | 222578 | 10 | 0 | 0.0189 | 0.0003 | 0.0184, 0.0195 |
| 29 | 222568 | 6 | 0 | 0.019 | 0.0003 | 0.0184, 0.0195 |
| 30 | 222562 | 19 | 4440 | 0.019 | 0.0003 | 0.0185, 0.0196 |
| 31 | 218103 | 461 | 0 | 0.0211 | 0.0003 | 0.0205, 0.0217 |
| 32 | 217642 | 373 | 0 | 0.0228 | 0.0003 | 0.0222, 0.0234 |
| 33 | 217269 | 349 | 0 | 0.0244 | 0.0003 | 0.0237, 0.0250 |
| 34 | 216920 | 242 | 0 | 0.0254 | 0.0003 | 0.0248, 0.0261 |
| 35 | 216678 | 207 | 0 | 0.0264 | 0.0003 | 0.0257, 0.0270 |
| 36 | 216471 | 275 | 0 | 0.0276 | 0.0003 | 0.0269, 0.0283 |
| 37 | 216196 | 253 | 0 | 0.0288 | 0.0004 | 0.0281, 0.0295 |
| 38 | 215943 | 233 | 0 | 0.0298 | 0.0004 | 0.0291, 0.0305 |
| 39 | 215710 | 257 | 0 | 0.031 | 0.0004 | 0.0302, 0.0317 |
| 40 | 215453 | 150 | 0 | 0.0316 | 0.0004 | 0.0309, 0.0324 |
| 41 | 215303 | 145 | 0 | 0.0323 | 0.0004 | 0.0316, 0.0330 |
| 42 | 215158 | 295 | 0 | 0.0336 | 0.0004 | 0.0329, 0.0344 |
| 43 | 214863 | 128 | 0 | 0.0342 | 0.0004 | 0.0334, 0.0349 |
| 44 | 214735 | 109 | 0 | 0.0347 | 0.0004 | 0.0339, 0.0354 |
| 45 | 214626 | 98 | 0 | 0.0351 | 0.0004 | 0.0344, 0.0359 |
| 46 | 214528 | 59 | 0 | 0.0354 | 0.0004 | 0.0346, 0.0362 |
| 47 | 214469 | 47 | 0 | 0.0356 | 0.0004 | 0.0348, 0.0364 |
| 48 | 214422 | 114 | 0 | 0.0361 | 0.0004 | 0.0353, 0.0369 |
| 49 | 214308 | 34 | 0 | 0.0363 | 0.0004 | 0.0355, 0.0370 |
| 50 | 214274 | 42 | 0 | 0.0364 | 0.0004 | 0.0357, 0.0372 |
| 51 | 214232 | 32 | 0 | 0.0366 | 0.0004 | 0.0358, 0.0374 |
| 52 | 214200 | 29 | 0 | 0.0367 | 0.0004 | 0.0360, 0.0375 |
| 53 | 214171 | 26 | 0 | 0.0368 | 0.0004 | 0.0361, 0.0376 |
| 55 | 214145 | 1 | 0 | 0.0368 | 0.0004 | 0.0361, 0.0376 |
| 60 | 214144 | 0 | 4717 | 0.0368 | 0.0004 | 0.0361, 0.0376 |
| 62 | 209427 | 492 | 0 | 0.0391 | 0.0004 | 0.0383, 0.0399 |
| 63 | 208935 | 138 | 0 | 0.0397 | 0.0004 | 0.0389, 0.0406 |
| 64 | 208797 | 36 | 0 | 0.0399 | 0.0004 | 0.0391, 0.0407 |
| 66 | 208761 | 1 | 0 | 0.0399 | 0.0004 | 0.0391, 0.0407 |
| 72 | 208760 | 1 | 0 | 0.0399 | 0.0004 | 0.0391, 0.0407 |
| 78 | 208759 | 1 | 0 | 0.0399 | 0.0004 | 0.0391, 0.0407 |
| 90 | 208758 | 0 | 4471 | 0.0399 | 0.0004 | 0.0391, 0.0407 |
| 120 | 204287 | 0 | 4698 | 0.0399 | 0.0004 | 0.0391, 0.0407 |
| 131 | 199589 | 22 | 0 | 0.04 | 0.0004 | 0.0392, 0.0408 |
| 150 | 199567 | 0 | 4553 | 0.04 | 0.0004 | 0.0392, 0.0408 |
| 180 | 195014 | 0 | 4717 | 0.04 | 0.0004 | 0.0392, 0.0408 |
| 210 | 190297 | 0 | 4510 | 0.04 | 0.0004 | 0.0392, 0.0408 |
| 240 | 185787 | 0 | 4458 | 0.04 | 0.0004 | 0.0392, 0.0408 |
| 270 | 181329 | 0 | 4405 | 0.04 | 0.0004 | 0.0392, 0.0408 |
| 300 | 176924 | 0 | 4084 | 0.04 | 0.0004 | 0.0392, 0.0408 |
| 330 | 172840 | 0 | 4382 | 0.04 | 0.0004 | 0.0392, 0.0408 |
| 360 | 168458 | 0 | 4797 | 0.04 | 0.0004 | 0.0392, 0.0408 |
| 390 | 163661 | 0 | 4755 | 0.04 | 0.0004 | 0.0392, 0.0408 |
| 420 | 158906 | 0 | 4515 | 0.04 | 0.0004 | 0.0392, 0.0408 |
| 450 | 154391 | 0 | 4242 | 0.04 | 0.0004 | 0.0392, 0.0408 |
| 480 | 150149 | 0 | 4435 | 0.04 | 0.0004 | 0.0392, 0.0408 |
| 510 | 145714 | 0 | 4218 | 0.04 | 0.0004 | 0.0392, 0.0408 |
| 540 | 141496 | 0 | 4145 | 0.04 | 0.0004 | 0.0392, 0.0408 |
| 570 | 137351 | 0 | 3910 | 0.04 | 0.0004 | 0.0392, 0.0408 |
| 600 | 133441 | 0 | 3860 | 0.04 | 0.0004 | 0.0392, 0.0408 |
| 630 | 129581 | 0 | 3574 | 0.04 | 0.0004 | 0.0392, 0.0408 |
| 660 | 126007 | 0 | 3711 | 0.04 | 0.0004 | 0.0392, 0.0408 |
| 690 | 122296 | 0 | 3379 | 0.04 | 0.0004 | 0.0392, 0.0408 |
| 720 | 118917 | 0 | 3908 | 0.04 | 0.0004 | 0.0392, 0.0408 |
| 750 | 115009 | 0 | 3832 | 0.04 | 0.0004 | 0.0392, 0.0408 |
| 780 | 111177 | 0 | 3539 | 0.04 | 0.0004 | 0.0392, 0.0408 |
| 810 | 107638 | 0 | 3388 | 0.04 | 0.0004 | 0.0392, 0.0408 |
| 840 | 104250 | 0 | 3277 | 0.04 | 0.0004 | 0.0392, 0.0408 |
| 870 | 100973 | 0 | 3161 | 0.04 | 0.0004 | 0.0392, 0.0408 |
| 900 | 97812 | 0 | 2984 | 0.04 | 0.0004 | 0.0392, 0.0408 |
| 930 | 94828 | 0 | 2904 | 0.04 | 0.0004 | 0.0392, 0.0408 |
| 960 | 91924 | 0 | 2685 | 0.04 | 0.0004 | 0.0392, 0.0408 |
| 990 | 89239 | 0 | 2554 | 0.04 | 0.0004 | 0.0392, 0.0408 |
| 1020 | 86685 | 0 | 2509 | 0.04 | 0.0004 | 0.0392, 0.0408 |
| 1050 | 84176 | 0 | 2284 | 0.04 | 0.0004 | 0.0392, 0.0408 |
| 1080 | 81892 | 0 | 2240 | 0.04 | 0.0004 | 0.0392, 0.0408 |
| 1110 | 79652 | 0 | 1987 | 0.04 | 0.0004 | 0.0392, 0.0408 |
| 1140 | 77665 | 0 | 2069 | 0.04 | 0.0004 | 0.0392, 0.0408 |
| 1170 | 75596 | 0 | 1805 | 0.04 | 0.0004 | 0.0392, 0.0408 |
| 1200 | 73791 | 0 | 1680 | 0.04 | 0.0004 | 0.0392, 0.0408 |
| 1230 | 72111 | 0 | 1697 | 0.04 | 0.0004 | 0.0392, 0.0408 |
| 1260 | 70414 | 0 | 1603 | 0.04 | 0.0004 | 0.0392, 0.0408 |
| 1290 | 68811 | 0 | 1568 | 0.04 | 0.0004 | 0.0392, 0.0408 |
| 1320 | 67243 | 0 | 1458 | 0.04 | 0.0004 | 0.0392, 0.0408 |
| 1350 | 65785 | 0 | 1387 | 0.04 | 0.0004 | 0.0392, 0.0408 |
| 1380 | 64398 | 0 | 1316 | 0.04 | 0.0004 | 0.0392, 0.0408 |
| 1410 | 63082 | 0 | 1267 | 0.04 | 0.0004 | 0.0392, 0.0408 |
| 1440 | 61815 | 0 | 1644 | 0.04 | 0.0004 | 0.0392, 0.0408 |
| 1470 | 60171 | 0 | 1327 | 0.04 | 0.0004 | 0.0392, 0.0408 |
| 1500 | 58844 | 0 | 1407 | 0.04 | 0.0004 | 0.0392, 0.0408 |
| 1530 | 57437 | 0 | 1273 | 0.04 | 0.0004 | 0.0392, 0.0408 |
| 1560 | 56164 | 0 | 1221 | 0.04 | 0.0004 | 0.0392, 0.0408 |
| 1590 | 54943 | 0 | 1203 | 0.04 | 0.0004 | 0.0392, 0.0408 |
| 1620 | 53740 | 0 | 1178 | 0.04 | 0.0004 | 0.0392, 0.0408 |
| 1650 | 52562 | 0 | 1134 | 0.04 | 0.0004 | 0.0392, 0.0408 |
| 1680 | 51428 | 0 | 1078 | 0.04 | 0.0004 | 0.0392, 0.0408 |
| 1710 | 50350 | 0 | 1021 | 0.04 | 0.0004 | 0.0392, 0.0408 |
| 1740 | 49329 | 0 | 1011 | 0.04 | 0.0004 | 0.0392, 0.0408 |
| 1770 | 48318 | 0 | 965 | 0.04 | 0.0004 | 0.0392, 0.0408 |
| 1800 | 47353 | 2 | 4.70E+04 | 0.0401 | 0.0004 | 0.0393, 0.0409 |
