# Supplementary material for: Parametric modeling of under-5 children survival among 30 African countries: Lognormal accelerated failure time gamma shared frailty model
Source: PLoS One. 2025 Jan 24;20(1):e0314955. doi: 10.1371/journal.pone.0314955 (PMC11759998; doi:10.1371/journal.pone.0314955)
Supplement: Attachment — Submitted filename: comment.pdf [file pone.0314955.s004.pdf]

Date: 14-sep-2024

General Comments

Thank you for inviting me to review a manuscript entitled *"**Modeling Survival Time to Death among Under-Five Children in Africa: Application of Lognormal AFT Gamma Shared Frailty Parametric Survival Regression Model**"*. This is an interesting study, and the authors have collected a good dataset using the critical advantage methodology. The paper is generally well written and structured. However, from my point of view, the manuscript has some shortcoming with respect to some dataset analyses and text, and I feel this good dataset has not been used to its full extent. Underneath, the reviewer has provided several remarks on the text, as it is often vague and long-winded. In several examples, I also recommended citing more relevant and recent literature. Additionally, the reviewer has made additional comments for more in-depth analyses of the dataset. I have assessed the manuscript, and I can hardly suggest it for publication for the following reasons:

Key critical points are:

a) Please check the grammar carefully in the overall manuscript; somewhere it's written in the past and somewhere in the present and somewhere it needs punctuations. Check the spelling mistakes; there are major spelling errors in the overall document. If possible, please I suggest that this manuscript be reviewed by an English-language expert.

Specific Comments

1. The **Background section on the body of manuscript**: this section needs revision so that it will cover the nature of the core problem, the known aspect of problem (i.e., what is known about the core problem), the unknown aspect of the problem (What is unknown about the

core problem), and the expectations from the current study (what and how the current study will add to the existing knowledge).

Abstract

Background

2. Is that true "Despite the significant progress made in reducing under-five mortality rates across African nations, the mortality rate in this age group continues to be the highest globally." What does it mean? Please write again?

Method

3. "This study employed a multivariable lognormal accelerated failure time gamma shared frailty parametric survival regression analysis to identify the predictors of time-to-death among infants in these African nations." Is this study for infants? Or for under five children?

Conclusion

4. In this study country region, maternal age, maternal education status, maternal age at first birth, respondent's employment status, birth outcome, wealth index, birth order, place of delivery, mode of delivery, women's autonomy in healthcare decision-making, number of antenatal care visits, child's size at birth, sex of the neonate, and community-level women's education. So, what? Please again and again and make a sense?

Method

5. The variables used in this study were extracted, cleaned, and processed using STATA version 14 software. Please remove it? This is repetition?
6. When we prepare manuscripts in Plos One no need of "**Keywords:** survival analysis, accelerated failure time model, Africa." Please read guide line for Plos One?
7. Please remove the citation style of (1), (2) of endnote style instead of this [1], [2]

8. Please use the latest reference rather than old reference? Like reference 2, 7, 9, 10, 49, 50, 51, 74 …etc…
9. How do you merge these 30 country datasets please give a clear justification?
10. Please include the STATA (version 14) commands that used for final model analysis as appendix (supplementary materials)?
